# Effects of 3 months continuous intake of supplement containing *Pantoea agglomerans* LPS to maintain normal bloodstream in adults: Parallel double‐blind randomized controlled study

**DOI:** 10.1002/fsn3.547

**Published:** 2017-11-20

**Authors:** Yoko Nakata, Chie Kohchi, Kazue Ogawa, Takeru Nakamoto, Hiroshi Yoshimura, Gen‐ichiro Soma

**Affiliations:** ^1^ Macrophi Inc. Takamatsu Kagawa Japan; ^2^ Department of Integrated and Holistic Immunology Faculty of Medicine Kagawa University Miki‐cho, Kida‐gun Kagawa Japan; ^3^ Non‐profit Organization, Linking Setouchi Innate Immune Network Takamatsu Kagawa Japan; ^4^ Central Park Clinic Takamatsu Kagawa Japan; ^5^ Nakagawa Hospital Fukuoka Japan; ^6^ Niigata University of Pharmacy and Applied Life Sciences Research Organization of Health for Self‐Reliance Niigata Japan

**Keywords:** arteriosclerosis, glycative stress, lipopolysaccharide, macrophage, vessel

## Abstract

In this study, the effects on the maintenance of normal bloodstream by lipopolysaccharide (LPS) were investigated in the parallel‐group randomized double‐blind study using supplement containing *Pantoea agglomerans* LPS (201.5 μg/tablet as LPS). Screening was previously performed in the implementation of the study. Adult males and females with normal value to borderline (healthy subjects) in the hematologic parameters, for which reference values were given, were chosen in this study. The period of ingestion of the supplement was 3 months. As the result, a significant decrease in the rate of change (the ratio when the baseline was 1) of HbA1c, which is a glycative stress marker, was found in the group which ingested LPS supplement after 3 months. Also, a significant increase in the number of fingertip capillary vessels was found compared with the control group. From these results, the effects of the maintenance of bloodstream by ingestion of LPS were shown.

## 1 INTRODUCTION

In Japan, with the declining birth rate and the aging of the population, from the viewpoint of the prolongation of healthy life expectancy and reduction in health costs, the importance of the self‐control of health by food ingestion is increasing.

Within this backdrop, LPS is one of the substances as a food ingredient that is expected to be useful for the maintenance of health (Inagawa et al., 1992). LPS, which is a cell wall component of Gram‐negative bacteria, acts by controlling macrophages which are the innate immune cells (Inagawa, Kohchi, & Soma, 2011). LPS is present in the environment by attaching to edible plants or floating in the air, and it has been recently revealed that the human immune system is controlled by ingesting LPS in the environment from diet or by breathing naturally (Braun‐Fahrländer et al., 2002). Since LPS has been found in Chinese medicines and edible plants, Inagawa et al. (1992) focused on the physiological actions of naturally ingested LPS, and the safety and effect of oral and transdermal ingestion were investigated by using LPS derived from Gram‐negative bacteria *Pantoea agglomerans* (LPSp).

In the study on the physiological actions of LPSp derived from *P. agglomerans*, (1) it was confirmed that stiff neck, malaise, lassitude, visual fatigue, and constipation were improved by the ingestion of LPSp combination *Citrus sudachi* juice for 3 weeks in 98 males and females aged from 30 to 60; 48.6 μg LPSp/day (open study) (Nakata et al., 2009). (2) In 47 males and females with high blood glucose levels and serum lipid levels aged from 40 years or older to 70 years or younger, it was confirmed that HbA1c levels and LDL marker were decreased by the ingestion of LPSp Salacia tea for 2 months; 500 μg LPSp/day (double‐blind study) (Nakata et al., 2011). (3) In 52 females aged from 40 to 79 years, it was confirmed that a decrease in the bone mineral density was suppressed by the ingestion of an LPSp soymilk for 3 months; 600 μg LPSp/day (double‐blind study) (Nakata et al., 2014). (4) In 20 males and females aged from 20 to 65 years, it was confirmed that triglyceride markers were decreased and malaise, lassitude, stiff neck, asthenopia, and constipation tended to improve by the ingestion of LPS beverage contained another *Pantoea* sp. for 1 month; 1200 μg LPS/day (open study).

Since these study results showed the safety of the oral ingestion of LPS and the usefulness in health maintenance from the improvement of stiff neck and asthenopia seen in (1) or (4), it is suggested that LPSp is more likely to promote improvement of the bloodstream.

LPS controls macrophages. Macrophages are known to produce nitric oxide (NO) with vasodilatory effects. Although the contribution of NO production by macrophages on vasodilatation has not been reported, there may be that possibility.

Also, macrophages function to eliminate unwanted molecules in the body by phagocytosis. From this point of view, there is the possibility that LPS eliminates intravascular degenerative foreign matter, for example, one of the AGEs, HbA1c, oxidized LDL, or triglycerides, by enhancing the phagocytosis of macrophages to contribute to improvement of the blood stream.

In addition, the bloodstream is associated with the number of capillary vessels, and it is known that macrophages produce vascular endothelial growth factor (VEGF) by stimulating with LPS (Cattin et al., 2015) (Spiller et al., 2014). The enhancement of VEGF production from macrophage and an increase in the number of capillary vessels by the ingestion of LPSp may lead to the improvement of the bloodstream.

In this study, in order to verify the functions in the maintenance of normal bloodstream of LPSp, HbA1c, which is one of the glycative stress markers, and oxidized LDL, which is one of the oxidative stress marker, the number of fingertip capillary vessels as well as safety markers were investigated by using the supplement containing LPSp alone and controls.

## 2 MATERIALS AND METHODS

### 2.1 Study design

This study was conducted as a randomized, parallel‐group, double‐blind study using reference products, and each parameter before, during, and after the ingestion of this test food was compared. Also, the LPSp supplement group and control group were compared. The study was conducted in accordance with the Declaration of Helsinki (1964) and Ethical Guidelines for Medical and Health Research Involving Human Subjects (22 December 2014, MEXT and MHLW), after being reviewed and approved by the Ethics Committee of Non‐profit Organization, Linking Setouchi Innate Immune Network (LSIN). All examinations were performed at LSIN collaborative medical facility (Miyake Medical Institute Group Central Park Clinic) under the control of the investigators. This study was registered in the University Hospital Medical Information Network (UMIN) Center (Registration Number: UMIN000019210).

**Figure 1:**
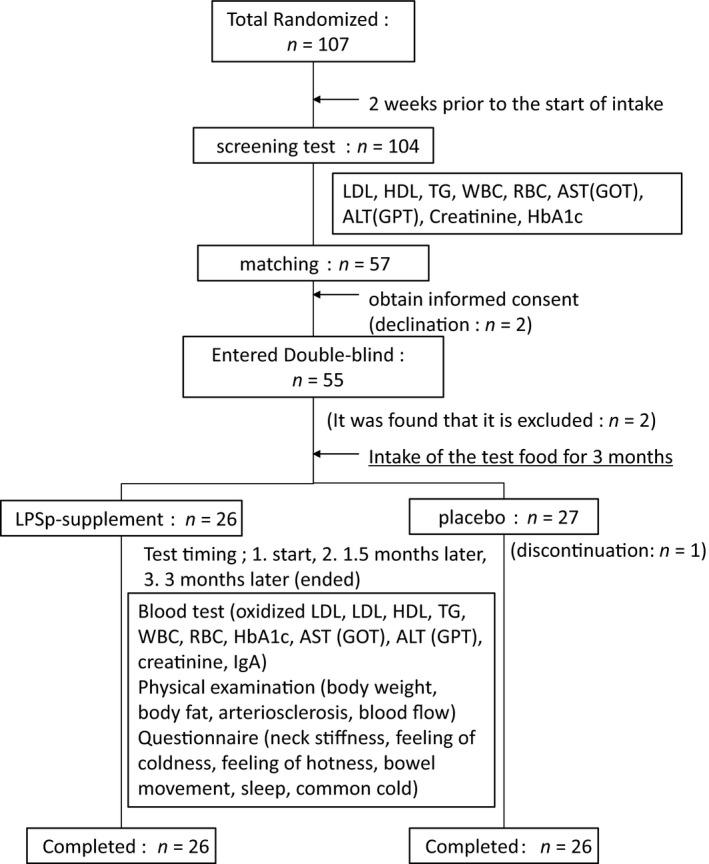
Study design and screening of subjects. Among the members enrolled in the LSIN, enrollment consent forms were obtained from persons who wished to participate in this study or persons who were newly recruited and consented to the enrollment. The content of the study was explained to 107 volunteers, the consent of the study purpose and the content was obtained, and the enrollment of this study was completed by obtaining the study consent forms at the same time (the informed consent was obtained). The screening was performed 2 weeks before the start of ingestion. Blood was collected from 104 subjects in the screening and a blood test was performed. The subinvestigator selected 57 subjects who met the inclusion criteria and did not conflict with the exclusion criteria based on the background of the subjects and the results of the blood test. Among these subjects, two subjects were declined to participate in the study before the start of the study and 55 subjects initiated the study

The study was conducted at the Miyake Medical Institute Group Central Park Clinic from 13 October 2015 to 5 February 2016, and the period of ingestion of the test food was 3 consecutive months. The grouping of the group which ingested the LPSp supplement and the group which ingested the controls was performed according to the envelope method, and this study was conducted according to the double‐blind approach. The study design is shown in Figure 1. Examinations were held four times at Central Park Clinic: at the screening, before the start of ingestion, after 1.5 months of ingestion, and after the completion of ingestion (after 3 months). The subjects were fasted after 9:00 p.m. on the day before examination.

### 2.2 Subjects

The subjects in this study included males and females aged 20 years or older and 74 years or younger, and subjects who were within the range of the hematological values before the start of the study excluding oxidized LDL, IgA, and CRP as shown in Table 1 (Mild abnormalities in the “Japan Society of Ningen Dock Criteria [Revised on 1 April 2014]”). In this blood test, the screening was performed prior to the start of the study. Other exclusion criteria were the following four items. (1) Subjects who are using the pharmaceuticals (pharmaceuticals used for the improvement of lipid metabolism or bloodstream) or foods (foods for specified health use or foods with function claims in which the improvement of lipid metabolism or bloodstream is clearly documented) that the investigator or the subinvestigator judged to have an impact on participation in the study. (2) Subjects who are participating or will be participating in another clinical study. (3) Subjects who have donated more than 400 ml of blood 3 months before the date of informed consent or more than 200 ml of blood 1 month before the date of informed consent. (4) Other subjects who are judged not to be eligible for participation in the study by the investigator or the subinvestigator.

**Table 1:** Range of each parameter

	Range of subject
LDL (mg/dl)	60–139
HDL (mg/dl)	40–119
TG (mg/dl)	30–199
WBC (×10^3^/μl)	3.2–8.9
RBC (×10^4^/μl)	Male: 400–599, female: 360–549
AST (GOT) (U/L)	0–35
ALT (GPT) (U/L)	0–40
Creatinine (mg/dl)	Male: up to 1.09, female: up to 0.79
HbA1c (%)	~5.9

WBC, white blood cell; RBC, red blood cell.

### 2.3 Test food

One tablet of this LPSp supplement compounds 25 mg of fermented wheat extract “Somacy‐FP100” produced by MACROPHI Inc. (Kagawa, Japan) and Somacy‐FP100 contains 10 mg/g of LPSp (measured value was 201.5 ± 22.8 μg/tablet by ELISA). For the control, the supplement replaced the LPSp of the LPSp supplement with dextrin. The name and mixing amount of the components of the test product are shown in Table 2.

**Table 2:** The name and mixing amount of components

Component	mg/tablet (rate)	
	LPSp supplement	Control
Somacy‐FP100(LPSp)	25.00 (12.5%)	0 (0%)
Maltitol	60.00 (30%)	60.00 (30%)
Dextrin	72.00 (36%)	97.00 (48.5%)
Cellulose	40.00 (20%)	40.00 (20%)
Calcium stearate	3.00 (1.5%)	3.00 (1.5%)
Total	200 (100%)	200 (100%)

Two tablets of LPSp supplement (403.0 μg LPSp/day) or control were ingested once daily before dinner in general. The supplement was dissolved by chewing or licking in the mouth and ingested in an unchanged form or with water.

### 2.4 Fermented wheat extract Somacy‐FP100

Somacy‐FP 100 is a raw material containing LPSp of *P. agglomerans*, a wheat symbiotic bacterium, as an active ingredient. Briefly, it is manufactured as follows. After *P. agglomerans* was cultured in a medium containing wheat flour as the carbon source, LPSp was extracted by heating and then purified by activated charcoal treatment, celite filtration, and membrane filtration. Finally, dextrin was added and spray dried so that the LPSp content was 10 mg/g to 15 mg/g. The components other than LPS contained in the raw material were as follows: moisture 4.7%, dextrin 89.9%, fiber 2.7%, protein 0.9%, lipid 0.5%, and ash content 0.3%. In addition, the wheat protein residue of Somacy‐FP 100 is “not detected.” On the other hand, it has been reported that various physiological activities, such as hyperlipidemia and allergy improvement, can be seen by ingesting LPS at 10 μg kg^−1^ day^−1^ (1 mg kg^−1^ per^−1^ day in terms of this raw material). The functional involved component is considered to be only LPSp.

### 2.5 LPSp measurement

LPSp was measured by sandwich ELISA using two kinds of mouse monoclonal antibodies (34G2 [IgM, κ], 4E11 ([gG, κ]) that specifically bind to the sugar chains of LPSp. Briefly, LPSp samples were added to a 96‐well immunoplate to which 34G2 was immobilized. After unbound contaminants were washed, 4E11 was added. After excess 4E11 was washed, alkaline phosphatase‐conjugated anti‐mouse IgG‐specific goat immunoglobulin (Sigma, Cat. No. 1418) was added to the wells. After washing, a colorimetric substrate, p‐nitrophenyl phosphate disodium solution, was added and the absorbance at 415 nm was measured with a microplate reader. As the standard, purified LPSp was used.

### 2.6 Endpoints

#### 2.6.1 Blood test

As the parameter that has an impact on the bloodstream, HbA1c was measured as a glycative stress marker. The lipid metabolism markers, such as LDL, HDL, triglyceride, and oxidized LDL, were measured at a time. The white blood cell count (WBC), red blood cell count (RBC), AST (GOT), ALT (GPT), creatinine, CRP, and IgA were measured as the safety markers. Measurements were held three times: before the start of ingestion, after 1.5 months of ingestion, and at the completion of ingestion (after 3 months). This blood test was analyzed by Shikoku Chuken Inc. (Kawaga, Japan).

#### 2.6.2 Vascular investigations

As direct investigations on the bloodstream and vessels, the investigation of arteriosclerosis (CAVI) and a vascular observation were performed. The right and left bloodstreams were measured by using the Fukuda Denshi Vascular Screening System “VS‐1500AN” for CAVI. The capillary vessel and bloodstream in the subungual space of the left ring finger were examined microscopically by using the Capillary Observation System of At Co., Ltd. for vascular observation; (1) drop the oil into the base of the claw of the left ring finger and place this finger on the measuring part (pedestal) of the microscope, (2) perform manual adjustment of the position or focusing of the microvessels, (3) save the obtained images on a PC (capture software), and (4) automatically quantify the length, thickness, turbidity, and horizontal line from the obtained image and then measure the number of blood vessels. And the images were transferred to the video device to analyze the vascular conditions. CAVI measurements were held three times: before the start of ingestion, after 1.5 months of ingestion, and at the completion of ingestion (after 3 months), and vascular observations were held twice: before the start of ingestion and at the completion of ingestion (after 3 months).

#### 2.6.3 Others

As a general physical examination, the body weight and percent of body fat were measured. In addition, as the evaluation for improvement in QOL, questionnaire surveys were administered on stiff neck, coldness, thermal sensation (hot flash), bowel movement, sleep, and common cold. Measurements of body weight and QOL surveys were held three times: before the start of ingestion, after 1.5 months of ingestion, and at the completion of ingestion (after 3 months). The visual analog scale (VAS) was also used for stiff neck and shoulder for QOL. On the scale of 10 cm with the left edge as the best and the right edge as the worst, the subjects marked the sensory position of the chronic symptoms on the examination date. An evaluation was made at the distance (mm) from the best point (left end) to the mark. For items other than shoulder stiffness, it was chosen from a given choice in a question paper for the presence and extent of subjective symptoms within the past week from the examination date.

### 2.7 Statistical analyses

By using statistical software (Excel Statistics 2012), the data quantified from the results of the blood test, investigation of arteriosclerosis, and vascular images were analyzed by the Steel‐Dwass test for intragroup comparisons and analyzed by the Mann–Whitney *U* test for intergroup comparisons. The statistical processing was carried out by excluding the data of subjects who were withdrawn or dropped out and missing (two‐sided significance level of 5%).

## 3 RESULTS

### 3.1 Subject

The study was started in 55 subjects through the screening. After the start of the study, a total of three subjects withdrew due to the following reasons: (1) the condition that should be excluded from the study was found after the start of the study, (2) subject wanted to withdraw due to the inconvenience of the examination schedule, and (3) the subinvestigator judged that it was difficult to continue the study through the interview. As a result, 52 subjects (26 in the LPSp supplement group and 26 in the control group) were eventually analyzed.

The ratio of male to female in each group was 9 males to 17 females in both groups. The mean age was 37.8 ± 7.2 years in the LPSp supplement group and 34.6 ± 9.9 years in the control group (Table 3).

**Table 3:** Baseline characteristics of the study population

	Control	LPSp supplement
*n*	26	26
Male	9	9
Female	17	17
Age (years)	34.6 ± 9.9	37.8 ± 7.2
Body weight (kg)	56.8 ± 11.8	56.6 ± 10.4
Body fat (%)	22.9 ± 5.3	22.9 ± 5.4

The numerical value which age, body weight, and body fat are *M* ± *SD*.

The body weight and percent of body fat at the start of ingestion were 56.6 ± 10.4 kg and 22.9 ± 5.4% in the LPSp supplement group and 56.8 ± 11.8 kg and 22.9 ± 5.3% in the control group, respectively. No significant changes in both body weight and percent of body fat were seen within and between groups (Table 4)

**Table 4:** Measurement of body weight and body fat

	Control			LPSp supplement		
	0 m	+1.5 m	+3 m	0 m	+1.5 m	+3 m
Body weight (kg)	56.8 ± 11.8	56.8 ± 11.7	57.4 ± 12.0	56.6 ± 10.4	57.0 ± 10.6	57.4 ± 10.4
Body fat (%)	22.9 ± 5.3	23.7 ± 5.8	24.9 ± 5.5	22.9 ± 5.4	23.5 ± 5.0	23.9 ± 5.4

Body weight and body fat were measured by ingested LPSp supplement or control. Subjects ingested test product for 3 months. Medical assessments were held three times; 0 m is baseline, +1.5 m is products ingested after 1.5 months, and +3 m is products ingested after 3 months. All numerical values are *M* ± *SD*.

### 3.2 Blood test

As the results of this study, according to the criteria as provided under the Japan Society of Ningen Dock Criteria (revised on 1 April 2014), the number of subjects with borderline or higher of each hematologic parameter was 1 or 2 subjects in the parameters of LDL, TG, WBC, ALT, creatinine, and CRP (Table 5).

**Table 5:** Transition of number of subjects with value within normal range and around borderline

				Control			LPSp supplement		
				0 m	+1.5 m	+3 m	0 m	+1.5 m	+3 m
Glucose metabolism marker	HbA1c (NGSP)	Normal range	<5.5%	22	22	23	22	21	23
		Borderline	5.6–5.9%	4	4	3	4	5	3
		Abnormality	>5.9%	0	0	0	0	0	0
	HbA1c (JDS)	Normal range	4.3–5.1%	6	7	6	2	2	5
		Borderline	5.2–5.8%	20	19	20	24	24	21
		Abnormality	>5.8%	0	0	0	0	0	0
Lipid metabolism marker	LDL	Normal range	60–119 mg/dl	21	20	19	22	22	20
		Borderline	120–139 mg/dl	5	6	6	4	3	4
		Abnormality	>139 mg/dl	0	0	1	0	1	2
	HDL	Normal range	40–119 mg/dl	26	26	26	26	26	26
		Abnormality	>119 mg/dl	0	0	0	0	0	0
	TG	Normal range	30–149 mg/dl	25	26	26	26	25	26
		Borderline	150–199 mg/dl	0	0	0	0	1	0
		Abnormality	>199 mg/dl	1	0	0	0	0	0
Safety maker	WBC	Normal range	32–85 × 10^2^/μl	25	26	26	26	26	26
		Borderline	86–89 × 10^2^/μl	0	0	0	0	0	0
		Abnormality	>89 × 10^2^/μl	1	0	0	0	0	0
	RBC	Normal range	Male: 400–539, female: 360–489 × 10^4^/μl	23	23	24	26	25	26
		Borderline	Male: 540–599, female: 490–549 × 10^4^/μl	3	3	2	0	1	0
		Abnormality	Male: >599, female: >549 × 10^4^/μl	0	0	0	0	0	0
	AST (GOT)	Normal range	0–30 U/L	25	26	25	25	25	25
		Borderline	31–50 U/L	1	0	1	1	1	1
		Abnormality	>50 U/L	0	0	0	0	0	0
	ALT (GPT)	Normal range	0–30 U/L	25	26	24	23	24	24
		Borderline	31–40 U/L	1	0	2	3	1	1
		Abnormality	>40 U/L	0	0	0	0	1	1
	Creatinine	Normal range	Male: <1.00, female: <0.71 mg/dl	25	26	25	25	24	24
		Borderline	Male: 1.01–1.09, female: 0.71–0.79 mg/dl	1	0	1	0	2	2
		Abnormality	Male: >1.09, female: >0.79 mg/dl	0	0	0	1	0	0
	CRP	Normal range	<0.31 mg/dl	26	25	26	25	26	26
		Borderline	0.31–0.99 mg/dl	0	0	0	1	0	0
		Abnormality	>0.99 mg/dl	0	1	0	0	0	0
Arteriosclerosis marker	CAVI^a^ right	Normal range	≥7.9	24	23	25	24	25	26
		Borderline	8.0–8.9	1	2	0	2	1	0
		Abnormality	9≤	0	0	0	0	0	0
	CAVI left	Normal range	≥7.9	23	23	25	24	25	26
		Borderline	8.0–8.9	2	2	0	2	1	0
		Abnormality	9≤	0	0	0	0	0	0

RBC, red blood cell; WBC, white blood cell. The transition of normal range and borderline number were surveyed by ingested LPSp supplement or control. Subjects ingested test product for 3 months. Medical assessments were held three times; 0 m is baseline, +1.5 m is products ingested after 1.5 months, and +3 m is products ingested after 3 months.

a One volunteer could not be measured after 3 months.

**Figure 2:**
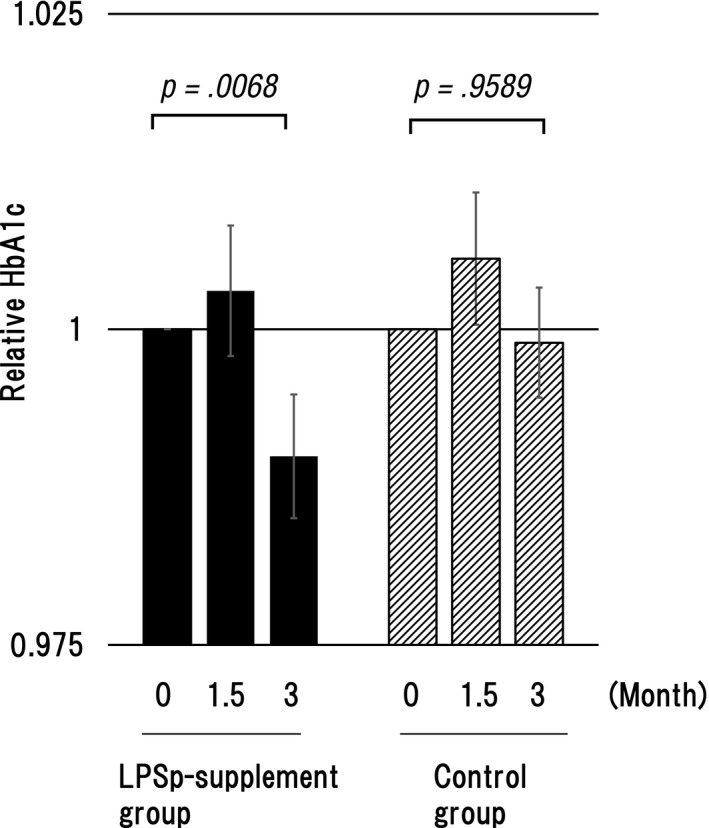
The changes in relative HbA1c levels in the LPSp supplement and control groups. HbA1c was measured before the start of ingestion (0), at 1.5 months, and 3 months after the start of ingestion. The graphic chart is expressed by *M* ± *SE* of the relative value in which the measured value before the start of ingestion is 1. P: Relative risk by Steel‐Dwass test (before the start vs. after 3 months of ingestion)

According to the JDS criteria, 24 subjects in the LPSp supplement group and 20 subjects in the control group were within the borderline at the baseline in HbA1c which is a glycative stress marker. Including the subjects with borderline, a significant decrease was found in the LPSp supplement group after 3 months of ingestion (Tables 5, 6, 7, Figure 2).

**Table 6:** Measurement of blood test

	Control			LPSp supplement		
	0 m	+1.5 m	+3 m	0 m	+1.5 m	+3 m
HbA1c (%)	5.3 ± 0.04	5.4 ± 0.06	5.3 ± 0.04	5.4 ± 0.03	5.4 ± 0.04	5.3 ± 0.04
Oxidized LDL (mg/dl)	96.7 ± 7.10	93.3 ± 3.94	98.3 ± 5.66	85.2 ± 4.58	97.4 ± 5.34	88.8 ± 6.25
LDL (mg/dl)	101.1 ± 4.99	100.0 ± 4.74	104.8 ± 5.00	100.0 ± 3.26	104.5 ± 3.21	104.4 ± 3.92
HDL (mg/dl)	64.9 ± 2.25	66.4 ± 2.54	66.3 ± 2.36	64.0 ± 2.54	66.3 ± 2.50	65.8 ± 2.55
TG (mg/dl)	71.1 ± 9.19	56.7 ± 4.70	64.9 ± 5.78	64.3 ± 4.30	64.9 ± 6.25	91.8 ± 25.62
WBC (×10^3^/μl)	54.4 ± 2.97	56.0 ± 3.54	54.0 ± 2.81	54.0 ± 2.35	54.4 ± 2.74	53.8 ± 1.80
RBC (×10^2^/μl)	460.2 ± 9.48	462.7 ± 7.40	466.4 ± 8.63	446.9 ± 7.29	457.1 ± 7.34	453.8 ± 7.26
AST (GOT) (U/L)	19.0 ± 0.82	19.2 ± 0.64	20.6 ± 1.14	19.1 ± 1.14	19.1 ± 1.20	20.8 ± 2.09
ALT (GPT) (U/L)	16.3 ± 1.30	15.5 ± 1.02	16.2 ± 1.48	16.7 ± 1.66	17.2 ± 2.07	17.0 ± 1.79
Creatinine (mg/dl)	0.64 ± 0.03	0.63 ± 0.03	0.63 ± 0.03	0.66 ± 0.03	0.65 ± 0.03	0.64 ± 0.03
CRP (mg/dl)	0.061 ± 0.01	0.098 ± 0.05	0.095 ± 0.03	0.067 ± 0.02	0.042 ± 0.01	0.040 ± 0.00
IgA (mg/dl)	228.2 ± 16.31	233.9 ± 16.82	231.5 ± 16.14	232.0 ± 20.09	237.0 ± 19.87	234.4 ± 19.66

RBC, red blood; WBC, white blood cell. Several items of blood test were measured by ingested LPSp supplement or control. Subjects ingested test product for 3 months. Medical assessments were held three times; 0 m is baseline, +1.5 m is products ingested after 1.5 months, and +3 m is products ingested after 3 months. All numerical values are *M* ± *SE*.

**Table 7:** Relative value of blood test

	Control			LPSp supplement		
	0 m	+1.5 m	+3 m	0 m	+1.5 m	+3 m
HbA1c	1.0 ± 0.0	1.006 ± 0.01	0.999 ± 0.00	1.0 ± 0.0	1.003 ± 0.01	0.990 ± 0.00^a^
Male	1.0 ± 0.0	1.018 ± 0.01	0.996 ± 0.01	1.0 ± 0.0	1.006 ± 0.01	0.996 ± 0.01
Female	1.0 ± 0.0	0.999 ± 0.01	1.001 ± 0.01	1.0 ± 0.0	1.001 ± 0.01	0.987 ± 0.01^a^
Oxidized LDL	1.0 ± 0.0	1.045 ± 0.057	1.068 ± 0.051	1.0 ± 0.0	1.165 ± 0.048	1.043 ± 0.047
LDL	1.0 ± 0.0	1.000 ± 0.028	1.047 ± 0.024	1.0 ± 0.0	1.056 ± 0.028	1.051 ± 0.030
HDL	1.0 ± 0.0	1.027 ± 0.020	1.025 ± 0.017	1.0 ± 0.0	1.051 ± 0.035	1.036 ± 0.024
TG	1.0 ± 0.0	0.901 ± 0.056	1.046 ± 0.077	1.0 ± 0.0	1.023 ± 0.069	1.301 ± 0.228
WBC	1.0 ± 0.0	1.067 ± 0.072	1.022 ± 0.051	1.0 ± 0.0	1.021 ± 0.037	1.019 ± 0.030
RBC	1.0 ± 0.0	1.009 ± 0.010	1.015 ± 0.008	1.0 ± 0.0	1.024 ± 0.009	1.016 ± 0.008
AST (GOT)	1.0 ± 0.0	1.034 ± 0.038	1.105 ± 0.057	1.0 ± 0.0	1.023 ± 0.051	1.072 ± 0.038
ALT (GPT)	1.0 ± 0.0	0.989 ± 0.044	1.032 ± 0.064	1.0 ± 0.0	1.066 ± 0.077	1.044 ± 0.047
Creatinine	1.0 ± 0.0	0.988 ± 0.013	0.986 ± 0.013	1.0 ± 0.0	0.986 ± 0.017	0.972 ± 0.015
CRP	1.0 ± 0.0	2.608 ± 1.621	2.685 ± 1.194	1.0 ± 0.0	1.154 ± 0.220	1.073 ± 0.175
IgA	1.0 ± 0.0	1.027 ± 0.013	1.020 ± 0.014	1.0 ± 0.0	1.027 ± 0.012	1.015 ± 0.013

RBC, red blood; WBC, white blood cell. The data of blood test were compared with the changes relative to the baseline. Subjects ingested test product for 3 months. Medical assessments were held three times; 0 m is baseline, +1.5 m is products ingested after 1.5 months, and +3 m is products ingested after 3 months. All numerical values are *M* ± *SE*.

a 0 m vs. +3 m, *p* < .01 by Steel‐Dwass test.

No significant changes in LDL, HDL, triglyceride, WBC, RBC, AST, ALT, creatinine, and CRP were found within and between groups in both the LPSp supplement and control groups, and these parameters were changed within the normal ranges (Tables 5, 6, 7).

The normal/borderline ranges were not specified in oxidized LDL and IgA, and no significant changes were found within and between groups in the measurement results (Tables 6 and 7).

No significant changes in the measured values of WBC, RBC, AST (GOT), ALT (GPT), creatinine, CRP, and IgA measured as safety markers in this study were found during the 3 months ingestion period, therefore it was confirmed that LPSp supplement was of no safety concern.

### 3.3 Bloodstream

No significant change in CAVI was found within and between groups in both the LPSp supplement and control groups, and this parameter was changed within the normal range. CAVI was significantly low in the control group at the baseline between groups, but no significant difference was found after 3 months (Table 8).

**Table 8:** Measurement of CAVI

	Control			LPSp supplement		
	0 m	+1.5 m	+3 m	0 m	+1.5 m	+3 m
Measurement						
Right	6.1 ± 0.18^a^	6.3 ± 0.17	6.4 ± 0.13	6.6 ± 0.16	6.5 ± 0.14	6.5 ± 0.16
Left	6.2 ± 0.18^a^	6.3 ± 0.17	6.4 ± 0.13	6.6 ± 0.14	6.5 ± 0.15	6.6 ± 0.15
Relative value						
Right	1.0 ± 0.0	1.050 ± 0.026	1.068 ± 0.029	1.0 ± 0.0	0.983 ± 0.015	0.995 ± 0.022
Left	1.0 ± 0.0	1.028 ± 0.025	1.044 ± 0.028	1.0 ± 0.0	0.989 ± 0.015	0.997 ± 0.018

CAVI of right body and left body were measured by ingested LPSp supplement or control. Subjects ingested test product for 3 months. Medical assessments were held three times; 0 m is baseline, +1.5 m is products ingested after 1.5 months, and +3 m is products ingested after 3 months. The date of CAVI was compared the changes relative to the baseline. All numerical values are *M* ± *SE*.

a Control 0 m vs. LPSp supplement 0 m, *p* < .05 by Mann–Whitney *U* test.

**Figure 3:**
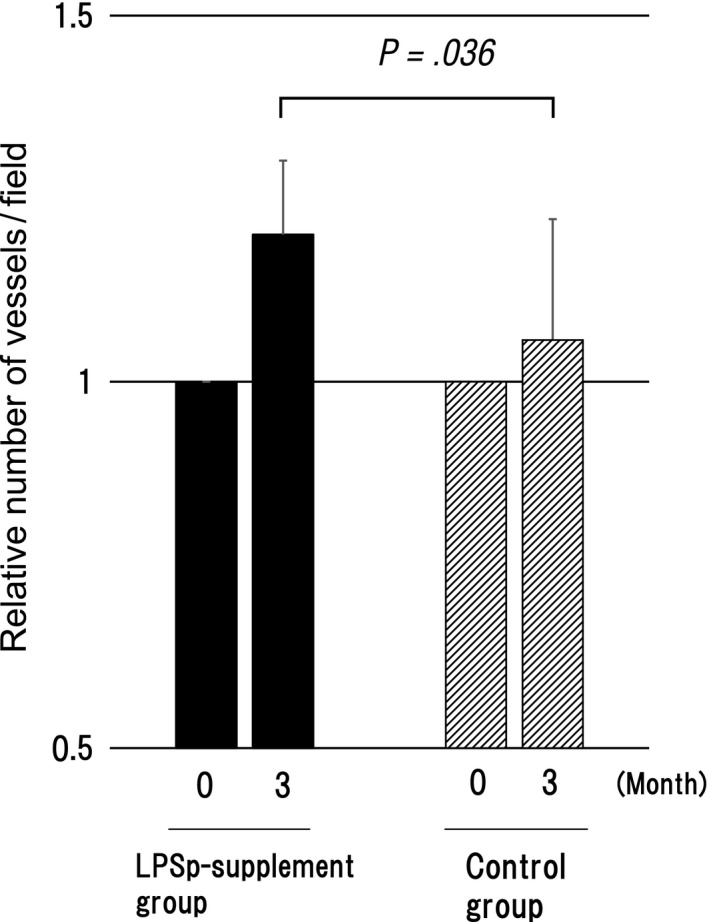
The changes in relative number of vessels per field in the LPSp supplement and control groups. The number of vessels was measured before the start of ingestion (0) and at 3 months after ingestion. The graphic chart is expressed by *M* ± *SE* of the relative value in which the measured value before the start of ingestion is 1. P: Relative risk by Mann–Whitney *U* test (test product group vs. control group)

By using the capillary bloodstream assessment system, when the number of fingertip capillary vessels that can be confirmed within the specified field was assessed as an indicator, a significant increase in the number of fingertip capillary vessels was found in the LPSp supplement group after 3 months compared with the control group (Table 9, Figure 3).

**Table 9:** Number of fingertip capillary vessel

	Control		LPSp supplement	
	0 m	+3 m	0 m	+3 m
Per field	4.92 ± 0.30	4.42 ± 0.25	4.65 ± 0.25	5.12 ± 0.27^a^
Relative value	1.0 ± 0.0	1.057 ± 0.17	1.0 ± 0.0	1.201 ± 0.10

Number of fingertip capillary vessel was measured by ingested LPSp supplement or control. It was observed in the area near lunula of the left ring finger. Subjects ingested test product for 3 months. Medical assessments were held two times; 0 m is baseline and +3 months is products ingested after 3 months. The date of capillary vessel number was compared with the changes relative to the baseline. All numerical values are *M* ± *SE*.

a Control +3 m vs. LPSp supplement +3 m, *p* < .05 by Mann–Whitney *U* test.

### 3.4 QOL

No significant difference in QOL was found in all parameters within the study period and between groups. However, in the LPSp supplement group, the subjects who developed stiff neck at the start of ingestion tended to improve after 3 months (Table 10). Also, the number of subjects who “did not feel coldness” was five at the start of ingestion, and the number of subjects doubled after 3 months for a total of 10 subjects (no data available).

**Table 10:** VAS of stiff neck

	Control			LPSp supplement		
	0 m	+1.5 m	+3 m	0 m	+1.5 m	+3 m
*M*	43.5 ± 6.52	35.6 ± 5.53	31.4 ± 5.66	60.4 ± 5.33	53.7 ± 7.37	42.7 ± 6.76
*p*						
0 m vs. +3 m	.173			.066		
Control +3 m vs. LPSp supplement +3 m	0.242					

VAS of stiff neck was researched by ingested LPSp supplement or control. Subjects ingested test product for 3 months. Medical assessments were held three times; 0 m is baseline, +1.5 m is products ingested after 1.5 months, and +3 m is products ingested after 3 months. All numerical values are *M* ± *SE*.

## 4 DISCUSSION

In this study, in order to verify the efficacy of the ingestion of LPS in the maintenance of normal bloodstream, the double‐blind study was conducted by the ingestion of LPSp supplement. Since this study assessed the functionality as the foods, it was conducted in healthy subjects and subjects with borderline.

LPS is known as an endotoxin that causes serious inflammation when it is released in the blood during the infection of Gram‐negative bacteria. On the other hand, LPS is a substance that is universally present in the environment. It is reported that it is not toxic by orally and transdermally ingested LPS, and it contributes to the activation of innate immunity.

LPSp used in this study is derived from wheat symbiotic bacteria *P. agglomerans*. This LPSp consists of rhamnose and glucose in the O‐antigen part, and is characterized by a shorter sugar chain length than that of *Escherichia coli* (Inagawa et al., 1992). The chromosomal aberration assay (1.5 mg LPSp/plate), bacterial reverse mutation test (1.5 mg LPSp/ml), single‐dose toxicity (600 mg LPSp/kg/day), and 28‐day repeated‐dose toxicity (300 mg LPSp/kg/day) have confirmed the safety of this LPSp (Taniguchi et al., 2009). In the results of this study, it was also confirmed that no adverse events were found in the healthy subjects and subjects with borderline with the oral ingestion of 500 μg/day LPSp.

In the bloodstream, Wiesner et al. (2010) reported that macrophage and intraperitoneally administered LPS, which is an activator of macrophage, may exacerbate arteriosclerosis. However, in this study, CAVI used as an indicator of arteriosclerosis was not changed in either group. Since the present study was performed on healthy volunteers, we cannot deny Wiesner's results, at least by oral administration; consequently, LPS may not be a factor in arteriosclerosis. No significant differences in other body markers and hematologic markers were found.

Here, we focused on the improved function of the bloodstream by the oral ingestion of LPSp. HbA1c is called advanced glycation end products (AGEs) in which hemoglobin and glucose are bound. In this study, 18.2% of subjects were included in the subjects with the borderline of HbA1c in both groups, respectively. A significant decrease (*p* = .007) in the rate of change in HbA1c was found in the LPSp supplement group after 3 months. This improvement was more significant for the female segment by gender. This may be because the number in (borderline for HbA1c in) the female segment was larger than that of the male segment. The decrease in HbA1c was consistent with the results of Nakata et al. (2011). Since macrophage distinguish denatured protein, such as AGEs, as a foreign matter and eliminate (Ott et al., 2014), the decrease in HbA1c may be related with phagocytic elimination by macrophages activated through oral administration of LPS. On the other hand, no change in oxidized LDL as an oxide marker was found in either group. It was considered that LDL in the subjects was within the normal range in both groups, in this study.

In association with the bloodstream, the number of fingertip capillary vessels was investigated in this study. Hypertensive patients showed lower capillary density, and capillary density was used for the assessment of antihypertensive drugs, etc. (Penna et al., 2008). Also, diabetic patients showed lower capillary density (Spiller et al., 2014). Since an increase in capillary density is associated with improvement of diseases (the effect of the uptake of glucose to tissues by insulin stimulation is dependent on the capillary vessel or the ability of oxygen diffusion to skeletal muscle tissue is enhanced), the capillary density is often used as an indicator of assessment. In everyday life excluding diseases, since the number of capillary vessels is decreased by stress or aging, antistress and the improvement of bloodstream can be assessed by investigating the number and status of capillary vessels.

In this study, an increase in the number of fingertip capillary vessels was found in the LPSp supplement group. As a result, it was newly suggested that LPSp promoted angiogenesis by stimulating the production of VEGF from macrophage and was likely to contribute to the improvement of bloodstream.

Moreover, IgA produced at mucosa was also measured as a secondary parameter in this study. This is because it has been reported that LPSp enhances the effect of the influenza vaccine when administered sublingually and promotes the systemic induction of IgA which is the mucosal immunity (Fukasaka et al., 2015). In this study, no increase in IgA was observed probably because there was no exposure to antigens during the study period.

Recently, it has been reported that orally and transdermally ingested LPS have a variety of effects on health maintenance and disease prevention. It has been reported that the oral ingestion of LPSp improved the alopecia status in mice that developed atopic dermatitis (Wakame, Komatsu, Inagawa, & Nishizawa, 2015), and it was also suggested that the gene expression of VEGF, when stimulated by LPSp, was increased in an LPSp dose‐dependent manner in human dermal papilla cell (Wakame et al., 2016). Also, it was confirmed that 62.5% (10 of 16 patients who were in remission or improved) of cancer patients were improved by the oral ingestion of LPSp (Morishima & Inagawa, 2016). As described in these reports, LPSp was found to exhibit effects on remission and the improvement of symptoms according to diseases.

From the results of this study, an increase in the number of capillary vessels and antiglycative stress effects by the oral ingestion of LPSp were found in the healthy subjects. Thus, it was suggested that orally ingested LPSp contributed to the maintenance of normal bloodstream. However, more study will be needed to determine the optimal dose of LPSp for the bloodstream.

## CONFLICT OF INTEREST

None declared.
